# Advances in High-Temperature Irradiation-Resistant Neutron Detectors

**DOI:** 10.3390/s25247554

**Published:** 2025-12-12

**Authors:** Chunyuan Wang, Ren Yu, Wenming Xia, Junjun Gong

**Affiliations:** Naval University of Engineering, Wuhan 430033, China; x24182703@nue.edu.cn (C.W.); dxaw110@sina.com (J.G.)

**Keywords:** high temperature neutron detection, Diamond detector, 4H-SiC detector, high temperature fission chamber, self-powered neutron detector

## Abstract

To achieve a substantial enhancement in thermodynamic efficiency, Generation IV nuclear reactors are designed to operate at significantly elevated temperatures compared to conventional reactors. Moreover, they typically employ a fast neutron spectrum, characterized by higher neutron energy and flux. This combination results in a considerably more intense radiation environment within the core relative to traditional thermal neutron reactors. Therefore, the measurement of neutron flux in the core of Generation IV nuclear reactors faces the challenge of a high-temperature and high-radiation environment. Conventional neutron flux monitoring equipment—including fission chambers, gas ionization chambers, scintillator detectors, and silicon or germanium semiconductor detectors—faces considerable challenges in Generation IV reactor conditions. Under high temperatures and intense radiation, these sensors often experience severe performance degradation, significant signal distortion, or complete obliteration of the output signal by noise. This inherent limitation renders them unsuitable for the aforementioned applications. Consequently, significant global research efforts are focused on developing neutron detectors capable of withstanding high-temperature and high-irradiation environments. The objective is to enable accurate neutron flux measurements both inside and outside the reactor core, which are essential for obtaining key operational parameters. In summary, the four different types of neutron detectors have different performance characteristics and are suitable for different operating environments. This review focuses on 4H-SiC, diamond detectors, high-temperature fission chambers, and self-powered neutron detectors. It surveys recent research progress in high-temperature neutron flux monitoring, analyzing key technological aspects such as their high-temperature and radiation resistance, compact size, and high sensitivity. The article also examines their application areas, current development status, and offers perspectives on future research directions.

## 1. Introduction

Nuclear energy occupies an important place in the global energy mix and has the advantages of high economy, sustainability, stability and zero carbon emissions. Recent advancements in nuclear reactor technology have led to the emergence of Generation IV reactors, which offer greater energy density, longer operational lifetimes, superior safety, and higher efficiency. Prominent examples include gas-cooled fast reactors, lead-cooled fast reactors, molten-salt reactors, sodium-cooled fast reactors, supercritical water-cooled reactors, and very-high-temperature gas-cooled reactors. To improve reactor thermodynamic efficiency, Generation IV reactors commonly use high-temperature coolants and new high-temperature-resistant structural materials, resulting in core temperatures that are much higher than those of conventional Generation III reactors. Representative temperatures include a coolant temperature of up to 650 °C in molten salt reactors [1], a core outlet temperature of 750–950 °C in high-temperature gas-cooled reactors [2], and a coolant outlet temperature of 500–600 °C in sodium-cooled fast reactors, with related nuclear vapor reaching 500 °C [3]. Generation IV reactors have abandoned the thermal neutron spectrum design of conventional light water reactors in favor of direct nuclear fission initiation using fast neutrons (energy > 0.1 MeV). Thus, fast reactors operate without moderators, which results in a longer neutron mean free path and a correspondingly higher neutron flux.

The combination of these attributes creates an exceptionally harsh environment within the core and on the internal surfaces of the pressure vessel, characterized by extreme temperatures and intense irradiation. High temperatures will lead to degradation of the thermal stability of traditional detector-sensitive materials, while high irradiation will trigger the accumulation of lattice damage and accelerate the failure of packages and functional components. Simultaneously, the synergistic effect of high temperature and intense irradiation causes significant degradation of the material properties in the sensitive regions of conventional detectors. This degradation leads to severe signal distortion or even complete obliteration by noise. Consequently, key performance metrics—including sensitivity, reliability, and operational lifetime—become inadequate, rendering traditional neutron detectors unsuitable for such applications.

To meet the needs of neutron flux measurements in Generation IV reactors, there is an urgent need to develop new high-temperature- and high-irradiation-resistant neutron detectors. Neutron detectors for high-temperature and high-irradiation environments have been extensively studied internationally, and the technical approaches adopted mainly include 4H-SiC neutron detectors, diamond neutron detectors, high-temperature fission chambers and self-powered neutron detectors. For the extreme environments of fourth-generation fast neutron reactors, four types of neutron detectors exhibit differing performance parameters and levels of technological maturity, as detailed in Table 1.

## 2. Diamond Detector

### 2.1. Working Principle

**Figure 1:**
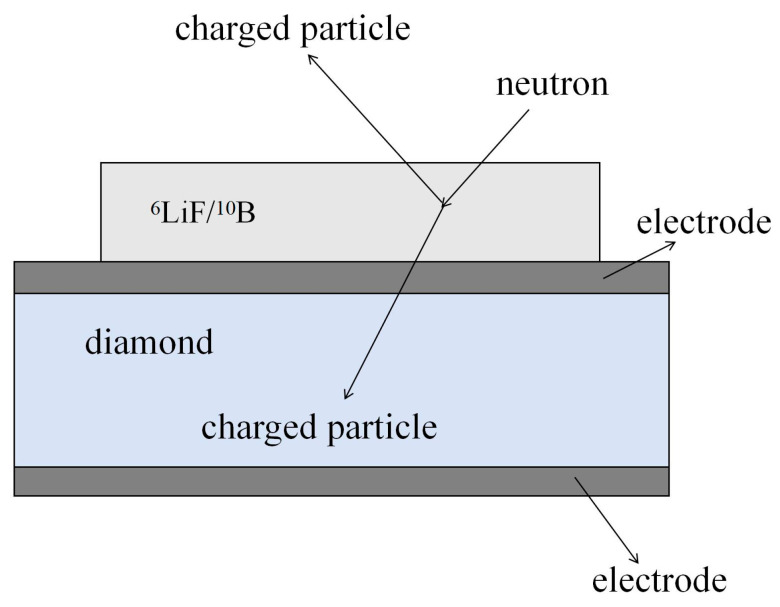
Schematic Diagram of the Basic Structure of a Diamond Detector.

Diamond material has the characteristics of large forbidden band width, high resistivity, high breakdown voltage, high atomic delocalization threshold energy, high carrier mobility, etc. Owing to their superior tolerance to high temperatures and irradiation, diamond detectors have emerged as a leading international research focus and a frontier in semiconductor-based nuclear radiation detection. As shown in Figure 1, a diamond detector generally consists of a diamond substrate, a neutron conversion layer, an electrode structure, an insulating and protective layer and a signal processing circuit, etc. The electrode structure usually consists of an ohmic contact or a Schottky contact formed by depositing metal on the diamond surface.

The operating principle of a diamond neutron detector is based on the interactions between incident fast neutrons and carbon-12 nuclei within the diamond lattice. These interactions, primarily elastic scattering ^12^C(n, n)^12^C, inelastic scattering ^12^C(n, n’)^12^C*, and nuclear reactions such as ^12^C(n, n’)3α and ^12^C(n, α)^9^Be, generate energetic secondary particles, including recoil nuclei and other charged particles. These secondary particles then deposit energy in the diamond material through ionization, creating electron–hole pairs which are collected as the detectable signal. A bias voltage is applied to both stages to create an electric field. Electrons and holes move towards the positive and negative electrodes, respectively, under the action of an electric field, forming a current signal that enables the measurement of the fast neutron flux.

The nuclear reaction method operates on the principle that a neutron is absorbed by a nucleus, triggering a nuclear reaction that emits charged particles. These particles subsequently cause ionization, generating the detectable signal. This mechanism is primarily employed for thermal neutron detection. Constrained by the reaction cross section of thermal neutrons with nuclides, nuclides such as ^10^B, ^6^Li, and ^3^He are commonly used in the nuclear reaction method with the following reaction equations:

(1) B510 + n → He24 + Li37 + 2.792 MeV,  σ = 3800 b

(2) Li36 + n → He24 + H13 + 4.786 MeV,  σ = 940 b

(3) He24 + n → H11 + H13 + 0.764 MeV,  σ = 5300 b

Due to its reaction threshold, diamond neutron detectors based on the ^12^C(n,α)^9^Be reaction (Equation (4)) exhibit limited efficiency for neutrons with energies below 6 MeV [4]. To circumvent this limitation, the incorporation of converter elements like ^6^Li or ^10^B into the diamond film has been proposed, which is expected to significantly enhance the detector’s detection efficiency. In this detection mechanism, low-energy incident neutrons interact with converter elements, generating secondary charged particles such as alpha particles and tritium nuclei. These particles then deposit energy within the diamond through ionization, promoting electrons to the conduction band and thereby creating electron–hole pairs. Under an applied electric field, these charge carriers drift toward the respective electrodes, where they are collected as an electrical signal. Thus, neutrons are measured indirectly by quantifying the resulting current or charge, which is proportional to the energy deposited by the charged reaction products.

(4) C612 + n → Be49 + He24

### 2.2. Preparation Process of Diamond Detectors

Diamond detectors have extremely strict requirements for diamond materials. Diamond materials applied to detectors should be characterized by high purity, single-crystal structure and low defect density. Single-crystal diamond is primarily available through artificial synthesis, given the rarity of its natural counterpart. The diamond material preparation process involves four main areas of research:

A critical initial step involves purification and impurity control, exemplified by techniques such as oxygen plasma purification. This process aims to achieve an ultra-high-purity material by reducing impurity concentrations to the parts-per-billion (ppb) level, thereby minimizing the detrimental trap effects within the crystal lattice. In 2019, a research team led by Jinlong Liu from the University of Science and Technology Beijing developed a high-purity single-crystal diamond detector [5]. They utilized oxygen plasma purification on commercial high-temperature high-pressure diamond seeds to control impurity incorporation down to the ppb level. The encapsulated detector was subsequently tested at the China Spallation Neutron Source, where it successfully demonstrated neutron pulse energy monitoring. However, the study did not include a systematic assessment of the detector’s performance parameters.

**Figure 2:**
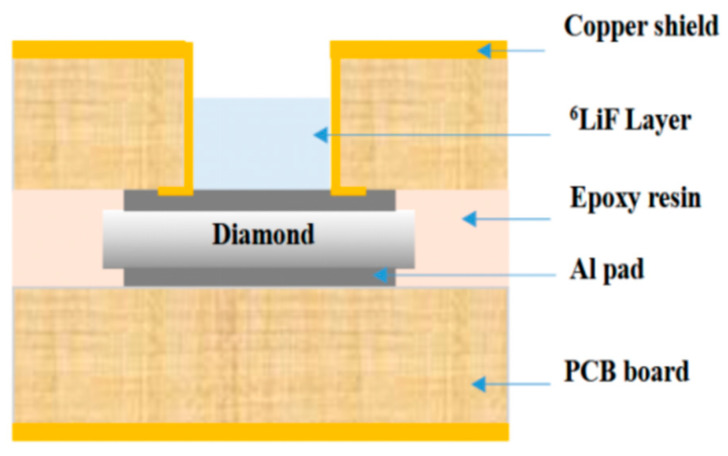
Schematic diagram of the internal structure of the diamond neutron detector [6].

Secondly, crystal growth technology primarily employs the microwave plasma chemical vapor deposition (MPCVD) method to produce high-purity, low-defect-density single-crystal diamond on high-temperature high-pressure substrates. The uniformity of the grown material can be further enhanced by optimizing the substrate holder design, for instance, through the implementation of groove structures. Liu Jiawei et al. from Wuhan University designed a novel grooved substrate holder for the MPCVD growth of single-crystal diamond using type IIa electronic-grade CVD diamond samples [6]. Based on this advancement, they fabricated a high-performance homoepitaxial diamond neutron detector, as illustrated in Figure 2. Characterization under alpha particle irradiation demonstrated that the detector exhibited an impulse response time of approximately 1.2 ns. The fabricated detector exhibited a response time approximately three times faster than that of a conventional silicon detector. Furthermore, it achieved an energy resolution of 3.7% and a charge collection efficiency of 97%. While the energy resolution is slightly lower, the charge collection efficiency is higher than that of a commercial single-crystal diamond detector under the same bias voltage. Overall, the device’s comprehensive performance is comparable to that of leading international commercial counterparts.

In 2024, Ding et al. [7] at Xi’an University of Science and Technology used the same method described above—MPCVD—to improve the detector performance again. A high-quality, 200 μm-thick single-crystal diamond was successfully synthesized on a high-temperature high-pressure substrate via homoepitaxial growth. The fabricated neutron detector demonstrated outstanding performance, achieving an energy resolution of 2.1% and a charge collection efficiency of 97.03% for electrons. This fabrication strategy shows significant potential to overcome the dual challenges of performance and cost associated with commercial diamond detectors.

This is followed by surface treatment and termination formation, including pre-treatment for impurity removal and the creation of oxygen terminations to optimize electrical characteristics. In 2025, Libin Wang et al. from Harbin Engineering University developed a high-performance oxygen-terminated single-crystal diamond detector [8]. The process began with the characterization and pretreatment of a 300 μm-thick diamond substrate to remove surface impurities and form an oxygen-terminated surface. Subsequently, metal electrodes were fabricated to establish high-quality ohmic contacts, resulting in the final device. The performance test results show that the energy resolution of the detector can reach 2.54%, the charge collection efficiency of electrons is as high as 98.9%, and the parameters such as the lower response limit are better than those of foreign commercial diamond detectors.

**Figure 3:**
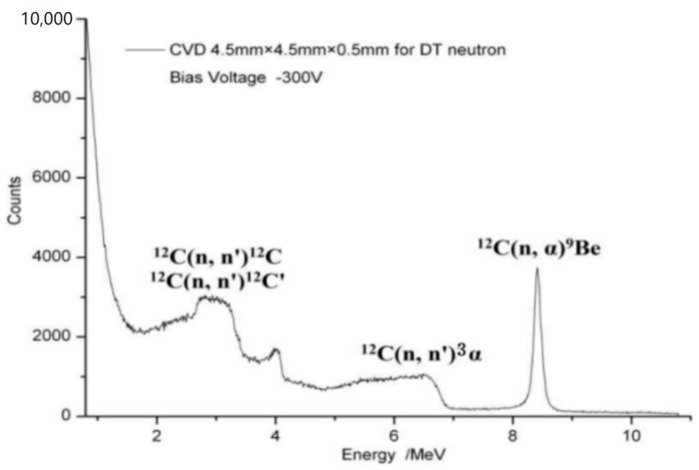
D-T fusion neutron energy spectrum measured by a diamond detector [11].

Finally, there is the electrode integration process, which improves the charge collection efficiency by preparing metal electrodes to achieve ohmic contact. Qingfeng Su et al. at Shanghai University systematically characterized the electrical and optical properties of diamond films, which guided the successful synthesis of detector-grade material [9]. On this basis, the Cr/Au double-layer electrode and annealing process were used to realize the ohmic contact between metal and diamond, and a diamond one-dimensional array particle detector was successfully developed. A CVD diamond thin-film detector for pulse amplitude measurements was developed using an opposite sandwich electrode structure with a homogeneous intrinsic diamond thin-film material by Wang Lan [10] and others at Tsinghua University. It can be used for fusion pulsed neutron and pulsed γ-ray measurements, with a dark current lower than 10 pA and a charge collection efficiency of 60–70%, which lays a good foundation for subsequent detector design and development. A diamond neutron detector for deuterium-tritium fusion neutron detection was developed by Xu Ping [11] et al. at Nanhua University, using a single-crystal diamond film with a thickness of 500 μm and a sandwich structure formed by a flat gold electrode and an orbital-shaped gold electrode. The characteristic peaks of ^12^C(n, a)^9^Be and ^12^C(n, n’)^3^α reaction can be detected, and the neutron energy spectrum is shown in Figure 3, with excellent energy resolution.

These fabrication processes share a common theoretical foundation centered on achieving high purity and low dislocation density. This principle guides the reduction in impurities and defects to optimize carrier transport, as well as the formation of ohmic contacts to enhance charge collection efficiency. Collectively, these measures ultimately improve key detector performance metrics, such as energy resolution.

### 2.3. Structural Design of Diamond Detectors

Neutrons with different energies exhibit distinct energy deposition characteristics in diamond. As electrically neutral particles, neutrons cannot directly deposit energy through ionization processes. Instead, they must undergo nuclear reactions or nuclear scattering to generate secondary charged particles, which are then detected by the diamond to achieve indirect neutron detection. For thermal neutrons, direct interaction with ^12^C is precluded due to the reaction threshold energy of ^12^C with neutrons. Therefore, isotopes such as ^6^Li and ^10^B are typically employed as neutron conversion materials, owing to their large thermal neutron reaction cross-sections. These materials react with thermal neutrons to produce high-energy charged particles, which subsequently generate electron–hole pairs upon entering the diamond. In the case of intermediate-energy neutrons, energy deposition occurs primarily through elastic scattering, resulting in the production of ^12^C recoil nuclei. For neutrons with energies exceeding 6.17 MeV, energy deposition is achieved via both elastic scattering and the nuclear reaction ^12^C(n,α)^9^Be. This process yields ^12^C recoil nuclei along with secondary charged particles, which collectively contribute to energy deposition within the diamond. Therefore, parameters such as the choice of material and thickness of the neutron conversion layer have a significant impact on the detection efficiency and sensitivity of the detector. At the same time, the structure of the detector, the diamond layer, etc., also determines the performance of the detector.

In terms of materials and structures, the neutron response is mainly enhanced by introducing ^6^LiF or ^10^B conversion layers. In 2017, the team of Marco Marinelli [12] developed a neutron detector with a ^6^LiF conversion layer using chemical vapor deposition of single-crystal diamond, which was tested in the TRIGA fission reactor with a power of 1 MW and a neutron flux of up to 2.2 × 10^9^ n/cm^2^/s, with a measured count rate of up to 1.5 × 10^5^ s^−1^, demonstrating the single-crystal diamond detector’s reliability in fission. The reliability of single-crystal diamond detectors for high flux neutron monitoring in fission reactors is demonstrated.

**Figure 4:**
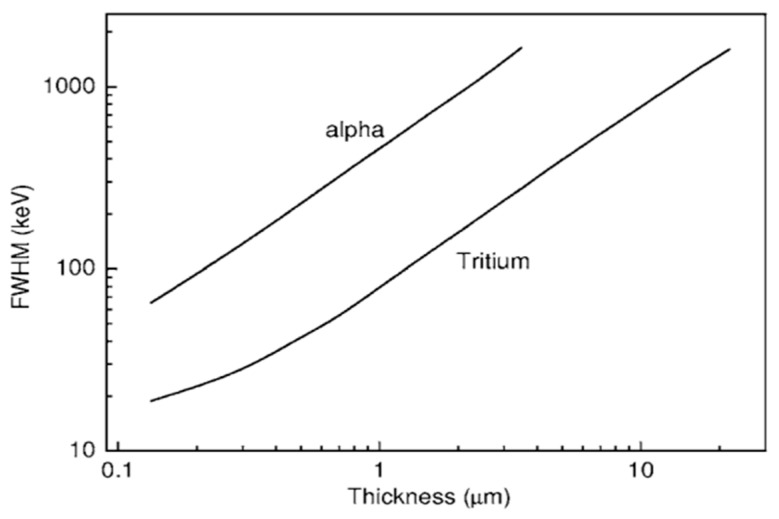
Half-height width of simulated 3H and ɑ peaks versus ^6^LiF conversion layer thickness [13].

The following year, also using chemical vapor deposition, the team of Almaviva S [13] developed a compact solid-state neutron detector capable of detecting both thermal and fast neutrons with a ^6^LiF conversion layer. The effects of the ^6^LiF conversion layer and diamond film thickness on neutron detection efficiency were systematically investigated. The results indicate that a thinner ^6^LiF layer improves the detector’s energy resolution but at the cost of reduced detection efficiency, revealing a critical trade-off between these two parameters. This relationship is further illustrated in Figure 4, which simulates the dependence of the FWHM of the tritium and alpha peaks on the ^6^LiF thickness, thereby providing valuable guidance for subsequent detector design.

The team of Tomoaki Masuzawa [14] carried out an in-depth to the mechanistic level study of the ^10^B conversion layer of diamond detectors. It first developed a polycrystalline diamond detector with a ^10^B-doped layer using hot filament chemical vapor deposition (HF-CVD). And then comparative experiments were carried out using the method of applying bias voltages to the top and bottom metal contacts with/without ^10^B-doped layers. It is demonstrated that the ^10^B-doped layer structure participates as a sensitive layer in the carrier transport of the detector and significantly improves the hole collection efficiency and hence the charge collection efficiency. Improvement of charge collection efficiency can be achieved in the future by developing detectors with multilayer structures.

In terms of signal processing, some international teams have used pulse shape screening (PSD) technology to distinguish neutron and γsignals, improving detection efficiency and signal-to-noise ratio. For low-energy neutrons, pulse shape discrimination (PSD) techniques can be employed to distinguish their signals from those generated by γ-rays. In the case of high-energy neutrons, once the recoil threshold is exceeded, discrimination between neutron energy deposition and γ-ray-induced signals can be more effectively achieved by analyzing differences in pulse amplitude. Beyond a specific energy threshold, the contribution from γ-rays becomes negligible, and energy deposition is dominated by neutrons. Consequently, the measured neutron energy spectrum typically exhibits a characteristic U-shaped distribution. In 2020, Makoto I. Kobayashi [15] et al. improved the detection efficiency of single-crystal CVD diamond detectors for thermal neutrons using PSD. The difference in the shape of the pulse formed by gamma rays and alpha particles was exploited to improve the thermal neutron detection efficiency by a factor of about 1.7.

**Figure 5:**
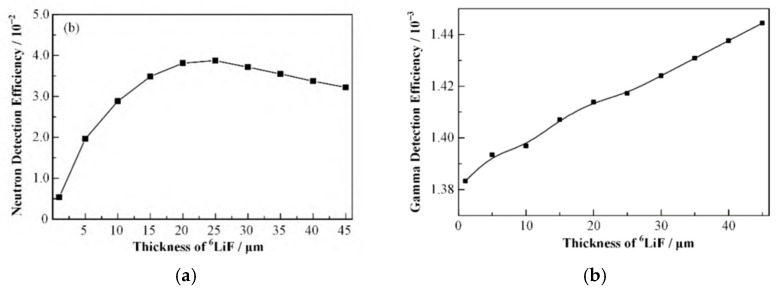
(**a**) Neutron detection efficiency at different ^6^LiF thicknesses [4]; (**b**) γ detection efficiency at different ^6^LiF thicknesses [4].

For performance simulation and optimization, researchers widely employ tools such as Monte Carlo codes and SRIM software (SRIM-2013). These enable systematic parametric studies on the influence of conversion layer thickness, diamond thickness, and discrimination thresholds on detection efficiency and the neutron-gamma discrimination ratio, thereby providing a theoretical foundation and clear pathways for detector design and optimization. Huan Ren [4] et al. at the University of Chinese Academy of Sciences used the Monte Carlo software (Geant 4) in combination with the SRIM software to simulate a diamond neutron detector. Calculation of the effects of neutron conversion layer (^6^LiF, ^10^B) thickness, diamond thickness, and γscreening threshold on the neutron detection efficiency, γdetection efficiency, and n/γsuppression ratio of the detector. The results are shown in Figure 5: the optimal thickness of the ^6^LiF conversion layer is 25 μm, and the diamond thickness needs to be greater than 20 μm, and the n/γsuppression ratio can be effectively improved by setting the γscreening threshold.

### 2.4. Performance Testing in High Temperature Environments

The demanding operating conditions of Generation IV reactors, characterized by extreme temperatures and intense radiation, necessitate neutron detectors capable of prolonged and stable operation. Conventional silicon-based detectors suffer severe performance degradation under such conditions, making it difficult to meet the demand. With its ultra-wide forbidden bandwidth, very high thermal conductivity, excellent resistance to irradiation, and good chemical stability, diamond is regarded as an ideal semiconductor detector material to replace silicon, and shows great potential especially in applications such as neutron detection. In recent years, a number of international research teams have conducted systematic and in-depth studies on the characteristics of diamond detectors for applications at high temperatures and in extreme environments, covering core aspects ranging from the selection of materials to the structural design of detectors, electrical performance at high temperatures, and long-term operational reliability. These studies have not only been rigorously tested under laboratory conditions, but also validated in real-world application scenarios of large-scale fusion devices such as JET, ITER-TBM, and the Scattered Fracture Neutron Source ISIS.

**Figure 6:**
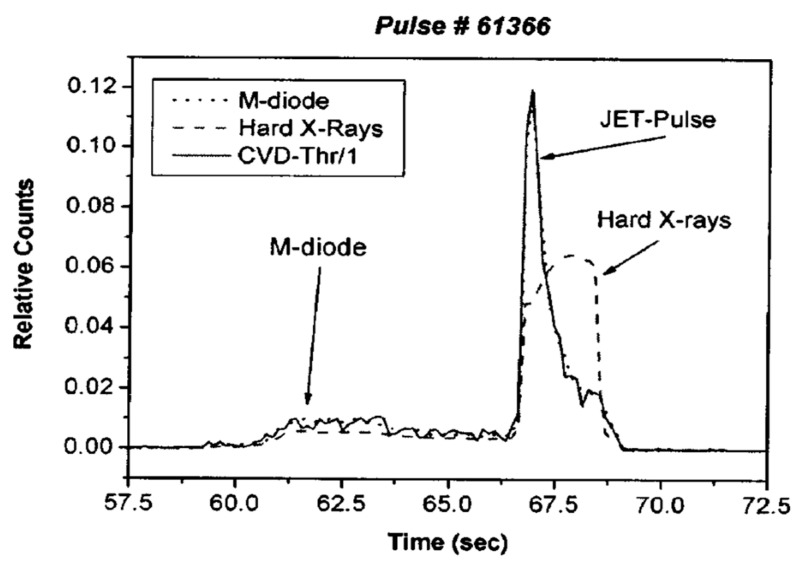
CVD diamond detector and M-diode silicon signals compared with hard X-ray signal for JET pulse No. 61366 [16].

For the application of diamond detectors in high temperatures and extreme environments, M. Angelone’s team has conducted systematic and comprehensive experimental studies and published a series of research results [16,17,18,19,20]. As early as 2005, in response to the serious degradation of the performance of traditional silicon detectors under high temperature and strong radiation environments, the team proposed the use of polycrystalline CVD diamond instead of silicon to develop polycrystalline diamond detectors. A benchmarking experiment was conducted at the JET fusion facility to compare the output signals of a diamond detector against those of a conventional silicon semiconductor detector. As shown in Figure 6, the diamond detector demonstrated suitability for neutron diagnostics at JET, exhibiting superior signal stability compared to its silicon counterpart.

**Figure 7:**
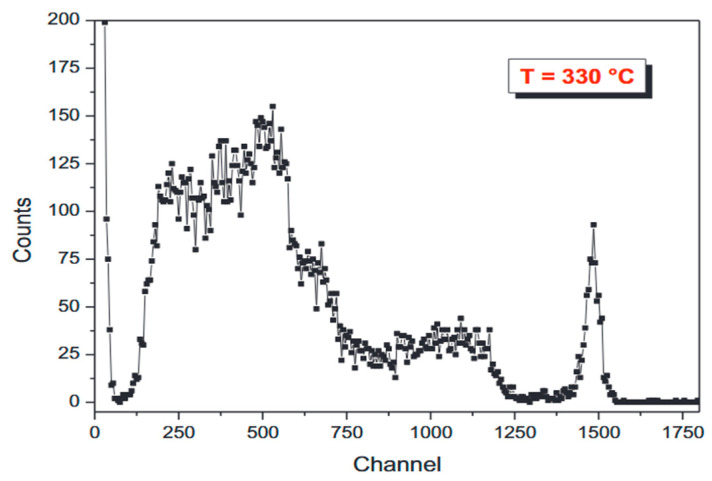
PHS recorded at 330 °C for the 100 mm thick diamond detector with the Cr-boron contact [18].

The team investigated the effect of operating temperature on the time dependence of a single-crystal diamond detector covered with a ^6^LiF conversion layer in 2013, and showed that the detector performance was not affected below 200 °C. And in 2016, the performance of ^6^LiF-coated monocrystalline diamond detectors at 190 °C was investigated. Preliminary demonstration of the feasibility of the detector as a monitor of tritium production in fusion reactors. At the same time, the chromium and silver contacts of the diamond detector have been experimentally investigated, and the metal contact, as the “conversion interface” between diamond and metal interface, plays an important role in the effective collection of electric charge. The experimental results show that the performance of silver contacts is better, but the metal contacts will be damaged under high temperatures and long-term operation. In the same year, in order to solve this problem, a new type of detector was developed with a Schottky-Chromium-Diamond metallic contact on one side and an ohmic non-metallic contact based on a heavily doped boron layer on the other side, which showed excellent performance at 300 °C. The pulse height spectrum is shown in Figure 7, where distinct characteristic peaks can be seen. In 2019, experiments on single-crystal diamond concluded that single-crystal CVD diamond detectors can operate stably up to 600 K in current mode, making them suitable for high-temperature, high-radiation environments such as ITER-TBM. The following year the team continued to investigate the effect of electrode contact on the high-temperature resistance of diamond neutron detectors. Experiments using three different types of electrical contacts, namely, dual Schottky contacts, dual ohmic contacts and Schottky-ohmic contacts, show that the detector using Schottky-ohmic contacts has better high-temperature resistance than those using the other two types of contacts, with a maximum operating temperature of 330 °C. The results show that the detector using Schottky-ohmic contacts has better high-temperature resistance than those using the other two types of contacts.

**Figure 8:**
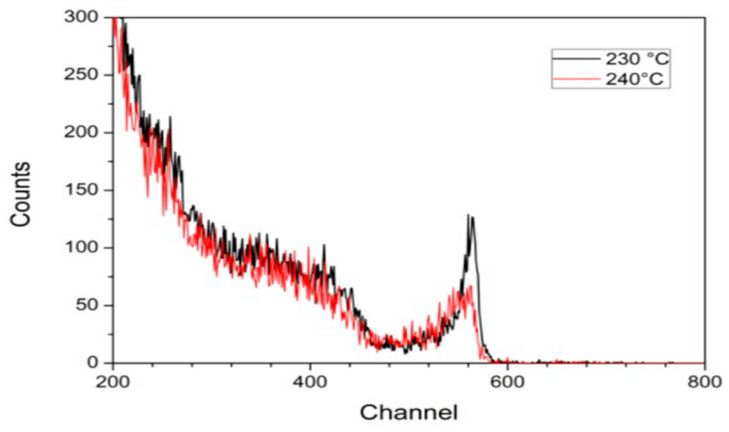
Comparison between PHS recorded at 230 °C and 240 °C. The degradation of the alpha peak is clear [22].

Masakatsu Tsubota’s team [21] investigated the electron transport properties of diamond detectors at high temperatures in 2015, analyzing and comparing three different types of diamond samples, and found that monocrystalline diamond has a better charge collection efficiency and stability at a temperature of 523 K, which makes it suitable for use in extreme environments. In 2016, R. Pilotti’s team carried out two studies at the Frascati Neutron Generator and the ISIS Scattered Neutron Source, respectively. The former [22] demonstrated that diamond detectors with silver contacts have good energy resolution at temperatures up to 235 °C, with a pronounced α-peak attenuation at 240 °C as shown in Figure 8. The latter [23] demonstrated that a diamond detector can be operated continuously for 100 h at high temperatures and high irradiation with a stability of up to 90%. In the same year, Muraro [24] et al. tested a matrix-type neutron detector consisting of 12 single-crystal diamond detectors on a JET and successfully reproduced the neutron energy spectrum. In 2018, Amit Kumar [25] et al. successfully demonstrated that single-crystal diamond detectors can operate at high temperatures of 300 °C. However, there are shortcomings: the detector’s encapsulation material needs to be further optimized to meet the higher temperature test requirements. In 2019, Pompili [26] et al. tested a diamond neutron detector on ITER and found no significant change in performance after two weeks of operation at 240 °C. Toyli Anniyev [27] and others in the same year carried out experimental tests on a high-purity diamond detector in the range of 25–175 °C, with a detector sensitivity area of 0.16 cm^2^. When the output of the pulsed neutron generator is 1.1 × 10^8^ n/s, the count rate of the diamond detector is 620 n/s, and the detection efficiency is 0.64%, which proves the close relationship between the detection efficiency and the area of the sensitive zone.

Overall, the stable operating temperature of diamond detectors is less than 300 °C. Exceeding this temperature threshold leads to progressive degradation in key detector performance metrics, including energy resolution, dark current, and charge collection efficiency. The primary cause is that elevated temperature induces thermal perturbations that disrupt carrier transport equilibrium. This occurs through two main mechanisms: firstly, a significant increase in dark current due to the thermal generation of electron–hole pairs; and secondly, the enhanced trapping of charge carriers at lattice defects such as vacancies and impurities. These effects collectively degrade charge collection efficiency and overall detector sensitivity. Therefore, the high purity of single-crystal diamonds is critical for their high-temperature detector applications.

### 2.5. Performance Testing in Irradiated Environments

For deployment in Generation IV nuclear systems, detectors must not only withstand high temperatures but also retain high performance and stability in intense radiation fields. In recent years, several studies have focused on evaluating the practical performance of diamond detectors in strong radiation fields. Together, these studies reveal the complexity of the evolution of diamond detector performance under harsh radiation conditions, providing an important basis for optimized design.

**Figure 9:**
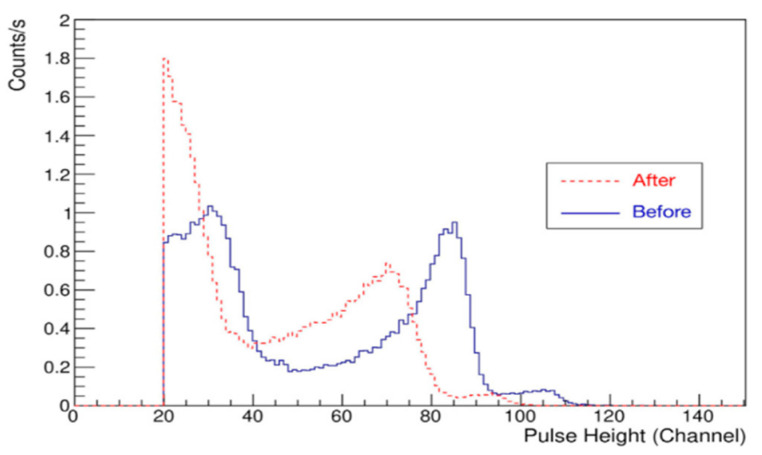
Irradiation effect. The pulse height distribution for detector in front of the thermal neutron beam before (solid line) and after (dashed line) irradiation by a 10^15^ n/cm^2^ fluence [30].

In terms of performance stability and strength, Liu [28] et al. of the China Institute of Atomic Energy Sciences (CIAE) investigated the performance of two different types of single-crystal diamond detectors under 100 MeV proton irradiation. The results show that the monocrystalline diamond detector can remain as a stable output signal after irradiation, but the signal output pulse height is reduced to 5% of the initial value. In terms of pulsed radiation field response characteristics, CIAE Ziye Wang [29] et al. conducted pulsed radiation field response characteristics tests on TW60019 single-crystal diamond detector using millisecond and nanosecond pulsed T-rays reference radiation field and a high-dose-rate γ irradiation device. The results show that the single-pulse response and collection efficiency of this model of diamond detector decrease with increasing pulse dose. To evaluate radiation hardness and charge collection characteristics, Jason Holmes et al. [30] characterized a diamond PIN diode detector employing a 1 MeV neutron flux of 10^15^ n/cm^2^. The corresponding results are presented in Figure 9. The neutron energy spectrum is shifted to the left by 250 kev after irradiation, but there is no significant change in the charge collection efficiency. To assess its comprehensive performance, Guangwei Huang et al. [31] evaluated a diamond neutron detector in a high-flux radiation environment. Their results demonstrated that the detection efficiency, energy resolution, and peak positions of the characteristic ^12^C(n,α)^9^Be reaction remained highly stable throughout the testing.

However, when evaluating the practical performance of diamond neutron detectors, key parameters beyond energy resolution and detection efficiency include the charge collection efficiency and its associated polarization effects. The charge collection efficiency governs the extent to which electron–hole pairs generated by radiation are converted into usable electrical signals. As demonstrated by Hodgson et al. [32], even high-quality single-crystal CVD diamond may exhibit polarization effects under intense α-particle irradiation due to the trapping of charge carriers at deep-level defects, which manifests as a drift in signal peak position over irradiation time. In the case of fast neutron detection, however, the influence of polarization is generally less pronounced than under α-particle irradiation, since recoil carbon nuclei and other secondary particles produce a more uniform ionization distribution within the detector bulk. Notably, incomplete charge collection does not entirely preclude neutron detection. Studies have shown that polycrystalline diamond detectors can maintain a stable counting response over a wide dynamic range of neutron flux even when the CCE is as low as ~4%. This makes them a viable alternative for applications that require cost-effectiveness, large-area coverage, or extreme radiation hardness. Consequently, in the selection of detectors for fourth-generation nuclear reactors, a comprehensive trade-off among material quality, long-term stability, and engineering feasibility must be carefully considered.

### 2.6. Short

Owing to their superior tolerance to both high temperatures and intense irradiation, diamond-based detectors represent a promising candidate for neutron monitoring in Generation IV nuclear energy systems. In order to achieve its reliable application in extreme environments, the research mainly focuses on material preparation, structural design and environmental adaptability. To illustrate the impact of different diamond materials and structural designs on detector performance, the representative findings from the aforementioned research teams are summarized in Table 2. This table compares the typical performance of single-crystal diamond detectors and polycrystalline diamond detectors across key performance parameters.

The fabrication process centers on producing high-purity, low-defect single-crystal diamond via MPCVD, followed by optimized surface termination and electrode processing. These processes collectively enable the detectors to achieve a charge collection efficiency exceeding 97% and an energy resolution better than 3%. Structurally, the introduction of ^6^LiF or ^10^B neutron conversion layers enhances neutron detection efficiency, while simulation-guided optimization of their thickness effectively improves the signal-to-noise ratio.

In terms of dealing with high-temperature environments, studies have confirmed that diamond detectors have excellent stability up to 300 °C, and designs such as the Schottky-Ohm hybrid contact can be used to further increase the operating temperature to 330 °C. The primary mechanism of performance degradation involves a sharp increase in dark current at elevated temperatures, coupled with carrier trapping by lattice defects. In terms of radiation resistance, diamond exhibits inherent robustness by maintaining stable signal output and satisfactory charge collection efficiency in strong radiation fields. Nonetheless, degradation in signal amplitude and spectral characteristics can occur following high-dose irradiation. This is particularly evident under transient pulsed radiation, where the detector’s response performance diminishes with increasing dose rate.

In summary, diamond detectors have become a more ideal choice for neutron detection in extreme environments. Future progress hinges on key advancements in material purification, defect engineering, and high-temperature encapsulation, coupled with a deeper understanding of irradiation damage mechanisms. These developments are essential for pushing the boundaries of the detector’s operational lifetime and application scope.

## 3. 4H-SiC Neutron Detector

### 3.1. Working Principle

**Figure 10:**
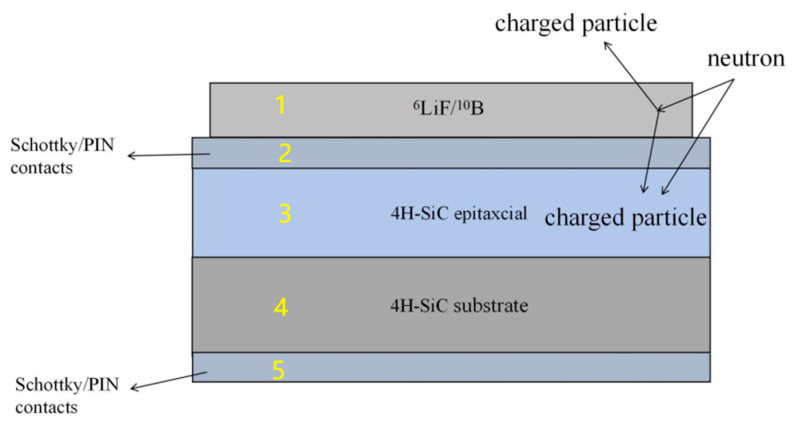
Basic Structure of 4H-SiC Neutron Detector.

4H-SiC neutron detectors are semiconductor detectors along with diamond detectors. The universal structural design is shown in Figure 10, which consists of five parts: neutron conversion layer, Schottky or PIN front contact, epitaxial layer, substrate and Schottky or PIN rear contact. The base material for the 4H-SiC neutron detector is the broadband semiconductor silicon carbide (4H-SiC). Doping the base material with different concentrations of elements such as nitrogen or phosphorus, usually low concentration doping for the epitaxial layer and high concentration doping for the substrate. A space charge region, which constitutes the depletion layer and acts as the sensitive volume of the detector, is formed at the PN or Schottky junction and resides almost entirely within the epitaxial layer. This region is depicted in Part 3 of Figure 10. In this region, the carriers are “swept away” to form a strong built-in electric field.

The operational principle of the 4H-SiC neutron detector relies on neutron interactions that generate secondary particles. These particles subsequently deposit energy within the sensitive region of the 4H-SiC material, enabling the detection of neutrons across varying energy levels. The deposition energy generates electron–hole pairs within the sensitive region, which in turn generate an electrical signal in response to a bias voltage [33]. For fast neutron measurement, the detection relies on direct nuclear reactions within the 4H-SiC sensitive volume, provided the neutron energy exceeds the specific reaction threshold. Principal reaction channels include ^12^C(n, n’)^3^α, ^12^C(n, α_0_)^9^Be, ^12^C(n, p)^12^B, ^28^Si(n, α)^25^Mg, and ^28^Si(n, p)^28^Al. For thermal neutron measurement, the neutron energy is typically below the threshold required for direct nuclear reactions. Similarly to diamond-based detectors, this necessitates the use of a conversion layer, typically composed of materials such as ^6^LiF or ^10^B_4_C, which is placed in front of the sensitive volume to facilitate the detection process. Through their reaction with the converter material, thermal neutrons produce secondary charged particles. These particles deposit energy in the sensitive region, thereby generating electron–hole pairs, which are then collected to produce an electrical output signal. The volume of the sensitive region directly affects the neutron detection efficiency, while the thickness of the epitaxial layer and the front contact determines how good the energy resolution of the detector is [34].

### 3.2. Preparation and Structural Design of Probes

For neutron detection in extreme environments, 4H-SiC emerges as a highly promising wide-bandgap semiconductor owing to its outstanding radiation resistance, excellent high-temperature stability, and high breakdown electric field. The structural design of the 4H-SiC detector, including the electrode contact form and the integration of the conversion layer, has a significant impact on the performance parameters such as neutron detection efficiency, energy resolution and signal-to-noise ratio. In recent years, concerted international research efforts have been dedicated to the systematic development of 4H-SiC neutron detectors, encompassing their design, fabrication, and performance optimization.

**Figure 11:**
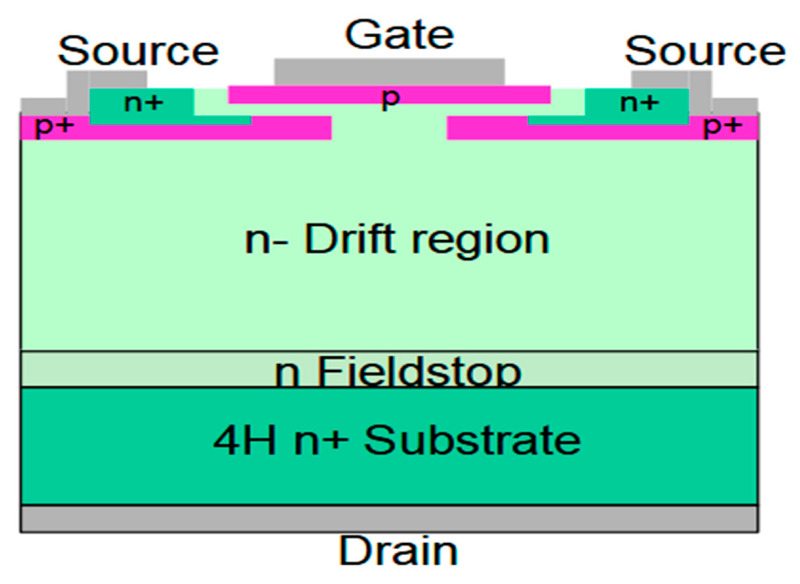
Schematic Structure of 4H-SiC Detector [39].

In 2012, a research team led by Yong Jiang at Sichuan University developed a neutron detector based on a 4H-SiC Schottky diode [35]. The device exhibited a low leakage current, maintained below 6.4 nA under reverse biases from 10 to 600 V, demonstrating the high quality of the Schottky contact. The neutron response of the detector to 5.486 MeV was also studied using a ^241^Am source, with a measured energy resolution of 4.5%. A team at the China Academy of Engineering Physics led by Chen Yu fabricated a 4H-SiC neutron detector with a ^6^LiF converter [36]. They characterized its alpha particle response using a ^241^Am source and its thermal neutron response in a critical assembly, finding a well-defined response and a linear relationship between count rate and reactor power. Separately, Haili Huang et al. combined established detector theory with semiconductor and circuit simulation methods to build a simulation model, facilitating the design and preparation of a 4H-SiC neutron detector [37]. Tingting Feng et al. at Xi’an University of Electronic Science and Technology designed a superjunction-modulated PIN diode neutron detector through multi-software simulations, also exploring key parameters such as charge collection efficiency and intrinsic detection efficiency [38]. Separately, Bohumir Zatko et al. developed a fast neutron detector based on a 4H-SiC epitaxial layer, as illustrated in Figure 11 [39]. The detector was used to measure fast neutrons from D–T reactions in the energy range of 16–18.3 MeV. An external high-density polyethylene (HDPE) conversion layer was incorporated to enhance detection efficiency.

The performance of 4H-SiC detectors is similarly constrained by their charge collection characteristics. In addition to the widely studied PIN or Schottky junction detectors based on high-quality epitaxial layers, semi-insulating bulk SiC has also been utilized for detector fabrication. Such materials typically exhibit lower CCE and more pronounced polarization effects. Their operating principle relies on the application of an electric field across the entire bulk material to collect charge, rather than solely depending on a depletion layer. Although their energy resolution is inferior to that of high-quality epitaxial devices, semi-insulating SiC detectors demonstrate favorable linear response and stability in fast neutron detection, along with relatively lower manufacturing costs and higher tolerance to bulk defects. This illustrates that, for certain reactor monitoring applications where flux counting rather than precise spectral analysis is required, a detector design offering compromised performance but greater robustness and cost-effectiveness can be adopted.

4H-SiC is a highly suitable semiconductor material for neutron detectors. Detectors fabricated from this material offer low noise, high energy resolution, and a linear response. A versatile detection capability, spanning thermal to fast neutrons, is achieved by incorporating appropriate conversion layers. This technology has now matured into a comprehensive framework encompassing device fabrication, performance testing, simulation-driven optimization, and practical deployment. Consequently, it demonstrates significant potential for applications in nuclear energy and technology, including reactor monitoring and fusion neutron diagnostics.

### 3.3. Performance Testing in High Temperature Environments

Facing the needs of neutron monitoring in high-temperature extreme environments such as nuclear reactors and fusion devices, the high-temperature stability of 4H-SiC detectors has become a research focus in recent years.

To systematically evaluate the high-temperature performance of 4H-SiC detectors, Zheng Li et al. pioneered a comprehensive test methodology [40]. They first constructed an integrated test system featuring precise temperature control, a vacuum environment, and electromagnetic shielding. Within this setup, the current-voltage (I–V) characteristics of the detector were measured across various temperatures using a high-precision current source. Concurrently, key performance parameters—including energy resolution, linearity, and charge collection efficiency for charged particles—were characterized using an α-source and a nuclear spectroscopy system. This approach effectively validated the detector’s electrical performance at elevated temperatures and its capability to detect charged particles, which simulate the secondary particles generated from thermal neutron interactions with a ^6^LiF conversion layer.

**Figure 12:**
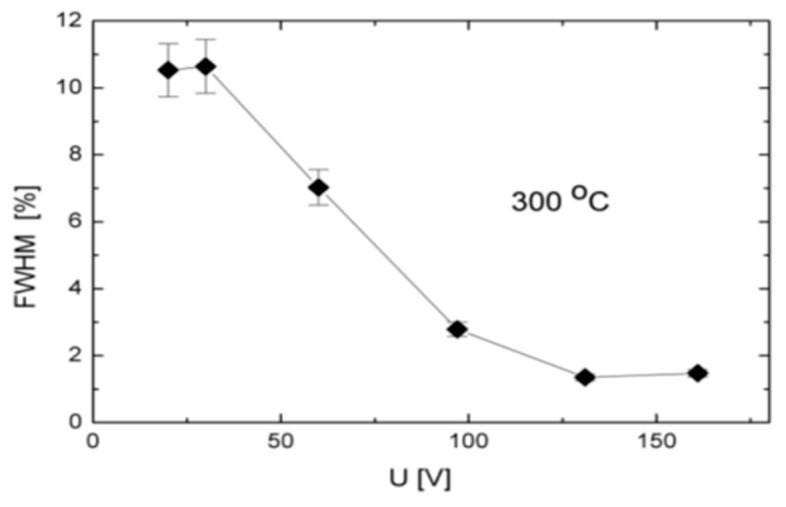
The energy resolution vs. bias voltage for a 4H-SiC detector [43].

International research has systematically identified the operational temperature limits of 4H-SiC devices and clarified the underlying mechanisms governing their high-temperature performance. Key limiting factors include a significant increase in thermal noise and leakage current, alterations in carrier transport behavior, the activation of intrinsic material defects, and the degradation of electrode contacts at elevated temperatures. These factors collectively determine the detector’s performance and stability in high-temperature environments. In 2006, Funaki et al. [41] demonstrated the superior high-temperature performance of Schottky barrier diodes (SBDs) relative to SiC JFET body diodes. In a separate study, Ivanov’s team [42] reported in 2009 that 4H-SiC PN junction detectors maintain an excellent energy resolution of up to 1.35% at temperatures as high as 300 °C. However, beyond 375 °C, bulk leakage currents induce a substantial noise increase. Conversely, an increase in the minority carrier diffusion length with temperature was observed, leading to a corresponding enhancement in the charge collection efficiency. A significant breakthrough was reported by Szalkai et al., whose 4H-SiC diode detector demonstrated stable detection of 14 MeV fast neutrons across a broad temperature range of 22–500 °C [43]. As shown in Figure 12, the device achieved an energy resolution of 2.2–2.3% at 300 °C under a 150 V bias and has been successfully deployed for characterizing high-temperature neutron flux in the ITER fusion reactor’s tritium breeding blanket. However, performance degradation occurs at 500 °C, primarily due to deterioration of the metal contacts; additionally, unadjusted bias at high temperatures exacerbates thermal noise. In 2024, Kushoro’s team [44] utilized the Frascati neutron generator to evaluate a 250-μm-thick 4H-SiC detector, employing high-temperature cabling and ceramic insulation for thermal management. Their experiments confirmed the stability of its key performance parameters at temperatures up to 250 °C, further underscoring the material’s exceptional temperature adaptability.

It is found that the 4H-SiC detector has better operational stability at high temperatures than the diamond detector. In 2022, C. Weiss [45] et al. tested the performance of 4H-SiC detectors with diamond detectors at high temperatures up to 500 °C. The results show that the charge collection efficiency of the diamond detector remains constant until 250 °C, after which there is a significant decrease. The dark current increases exponentially with temperature and the noise level is constant over the entire temperature range. The increasing drift time of the electrons with temperature leads to a constant charge collection efficiency of the 4H-SiC detector until 350 °C, which decreases by 70% at 500 °C.

### 3.4. Neutron Monitoring Applications in Reactors

In the fields of nuclear reactor monitoring and fusion neutron diagnostics, the engineering application of 4H-SiC neutron detectors is transitioning from the proof-of-principle stage toward practical system integration.

**Figure 13:**
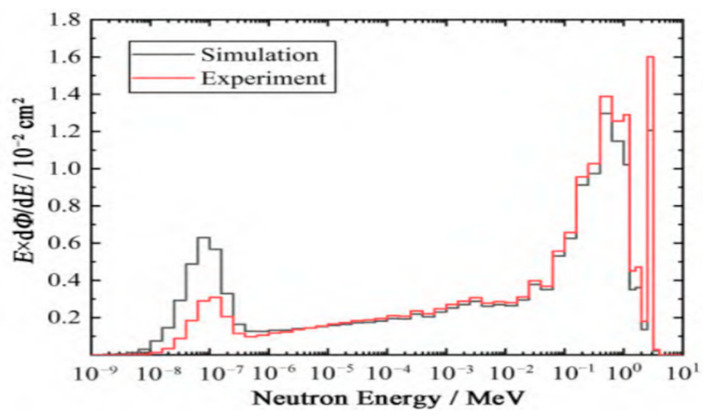
Comparison between simulated and experimental energy spectra at the measurement position [47].

Researchers at the University of Science and Technology of China have validated the core performance of 4H-SiC detectors using an intense deuterium-tritium fusion neutron source. Wang Feipeng et al. confirmed the detector’s excellent energy resolution and signal stability [46]. In a parallel development, the team led by Mingqiang Zhang innovated the design of a Bonner spectrometer system by integrating a ^6^LiF-coated 4H-SiC detector with a moderated sphere array [47]. This system successfully monitored the neutron energy spectrum and dose equivalent during neutral beam injection experiments at the EAST Tokamak. The experimental results, presented in Figure 13, show strong agreement with simulation data.

Internationally, parallel research efforts are underway. Radulović’s team [48] evaluated 4H-SiC detectors with sensitive volumes of 25–170 μm for 0–5 eV thermal neutron detection in the TRIGA reactor at 240 °C. The detectors demonstrated sensitivity comparable to conventional BF_3_ and ^3^He detectors, establishing a foundation for their use in multi-detector arrays. The most systematic breakthrough in this field stems from a decade-long research program led by Frank H. Rudd’s team. Their work began in 2002 with the pioneering integration of a 4H-SiC detector into the inner wall of the IRIS light-water reactor for stable monitoring of neutrons above 1 MeV under harsh conditions (fast neutron flux: 1.6 × 10^11^ n/cm^2^/s, 292 °C) [49]. This was followed in 2003 by the development of an iterative inverse convolution method for energy spectrum unfolding, leveraging response functions modeled with SRIM/MCNP and validated against mixed-field sources including a D-T neutron generator and ^252^Cf [50]. By 2005, the team had designed a detector array for full-scale reactor power monitoring and confirmed the stability of the spectral response at 306 °C using a ^240^Pu neutron source [51]. Subsequent research overcame critical challenges such as multi-peak resolution for 14 MeV fast neutrons [52] and the detection of thermal neutron-induced fission [53]. A notable milestone was achieved when distinct reaction peaks from ^12^C(n,α)^9^Be and ^28^Si(n,α)^25^Mg were clearly resolved at an extreme temperature of 500 °C [54]. Collectively, these efforts have established a comprehensive technical framework for energy spectrum measurement and high-temperature online monitoring with 4H-SiC detectors in complex radiation fields, providing vital support for the intelligent sensing needs of advanced nuclear energy systems.

Radulovic et al. [55] reported on a study of silicon carbide (4H-SiC) neutron detectors conducted by the E-SiCure collaboration at the JSI TRIGA reactor. The study systematically investigated radiation-induced defects in 4H-SiC Schottky diodes through neutron irradiation experiments. The team further fabricated 4H-SiC detector prototypes incorporating ^10^B and ^6^LiF conversion layers. These prototypes successfully detected thermal neutrons in a reactor neutron field, demonstrating the viability and stability of 4H-SiC detectors in radiative environments and thereby providing crucial experimental support for the development of future portable and radiation-tolerant neutron detectors. Mandal et al. [56] conducted a systematic investigation of Schottky barrier neutron detectors based on high-quality 4H-SiC epitaxial layers, demonstrating their potential for applications in harsh environments such as reactor dosimetry. The researchers prepared n-type 4H-SiC epitaxial layers with thicknesses ranging from 20 to 250 μm using the chemical vapor deposition (CVD) method, and fabricated Ni/4H-SiC detectors exhibiting a high Schottky barrier height (~1.6 eV) and low leakage current. In α-particle detection experiments, the detectors achieved an energy resolution of 0.29%, comparable to that of silicon-based detectors. Furthermore, the authors examined the effect of neutron irradiation on detector performance, revealing that the devices remained functional even after exposure to a neutron fluence of up to 5 × 10^13^ cm^−2^, with only a moderate degradation in energy resolution. Kandlakunta et al. [57] addressed the critical requirements of radiation and high-temperature tolerance for neutron detectors used in near-core high-flux neutron monitoring. Focusing on 4H-SiC as the core material, they conducted systematic experiments within the graphite thermal column of the Ohio State University Research Reactor (OSURR). The research team positioned custom-fabricated 4H-SiC Schottky diode detectors at three key locations in the thermal column, collecting energy spectra and raw waveform data under mixed n–γ fields across reactor power levels ranging from 100 to 450 kW. The experimental results demonstrated that the 4H-SiC detectors exhibited an excellent linear response to steady-state reactor power. The power values estimated from the detector signals showed strong agreement with data from standard compensated ionization chambers, and the detectors accurately mapped the relative neutron flux at different near-core positions, with errors of less than 5%. Furthermore, no significant performance degradation was observed in the 4H-SiC detectors under high-radiation conditions, confirming their radiation hardness and suitability for long-term reactor monitoring applications.

### 3.5. Short

Substantial progress has been achieved in the development of 4H-SiC neutron detectors, encompassing structural design, high-temperature performance, and in-reactor applications.

The design and preparation of 4H-SiC neutron detectors have evolved toward greater diversity and sophistication. While early work centered on verifying the performance of basic device architectures—such as the low-leakage Schottky and PN junction diodes developed by teams from Sichuan University and CAEP—a critical advancement has been the functional integration of neutron conversion layers. These include ^6^LiF coatings for thermal neutron sensitivity and high-density polyethylene (HDPE) to improve fast neutron detection efficiency.

Secondly, significant and systematic progress has been made in adapting these detectors to high-temperature environments, spanning from standardized performance evaluation to breakthroughs in operational limits. To address the monitoring requirements of nuclear reactors and fusion devices, researchers have established a standardized high-temperature testing methodology that integrates precise temperature control, vacuum conditions, and electromagnetic shielding. Through continued experimental investigation, the upper operating temperature limit of these detectors has been progressively elevated, with state-of-the-art devices now capable of functioning at temperatures up to 500 °C.

Finally, in reactor monitoring applications, the transition from single-point detection to comprehensive system-level monitoring is being progressively realized. The maturing technology has now been successfully deployed in several nuclear facilities. Illustrative examples include the work by a team from the University of Science and Technology of China, who innovatively coupled a ^6^LiF-coated 4H-SiC detector with a Bonner sphere spectrometer on the EAST tokamak, enabling real-time neutron energy spectrum and dose monitoring. Teams including Radulovic, Mandal, and Kandlakunta have validated the feasibility and stability of their self-developed 4H-SiC detectors under irradiation conditions within an actual reactor neutron field. The most representative long-term effort is the multi-decade research by Frank H. Rudd’s team. Their systematic contributions include the integration of detectors into light-water reactor pressure vessels, the development of a response function model coupled with an iterative deconvolution method for accurate spectrum unfolding, and the creation of detector arrays for full-scale reactor power monitoring. Notably, their detectors can clearly resolve characteristic nuclear reaction peaks at extreme temperatures up to 500 °C. Collectively, this work has established a complete technical framework for online monitoring in complex radiation environments.

## 4. High Temperature Fission Chamber

As the core equipment for neutron flux monitoring in nuclear reactors, the technological development of fission chamber detectors has always been closely centered on the needs of the high-temperature and high-radiation environment of nuclear energy systems. With the rise of fourth-generation nuclear reactors (e.g., sodium-cooled fast reactors, high-temperature gas-cooled reactors), the extreme high-temperature conditions within the core have posed unprecedented challenges to the high-temperature resistance of detectors, driving the breakthrough of fission chamber technology from traditional design to two major directions: high-temperature adaptability and miniaturized structure.

This chapter systematically reviews key technological advances in fission chamber detectors, focusing on their high-temperature performance, deployment in reactor monitoring, miniaturized structural design, and performance optimization.

### 4.1. Working Principle

**Figure 14:**
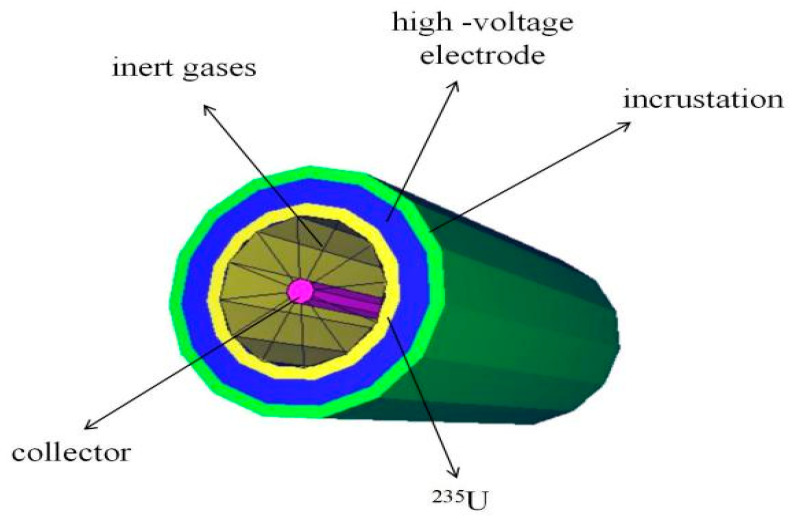
Schematic diagram of the basic structure of a fission chamber.

A high-temperature fission chamber, illustrated in Figure 14, is typically composed of fissile material coatings, electrodes, an inert fill gas, electrical insulators, and a sealed housing. Its operation begins when incident neutrons interact with fissile coatings, such as ^235^U or ^238^U [58], applied to the cathode, inducing nuclear fission and generating energetic fission fragments. These heavy, charged fragments are the primary source of the detector signal. They ionize the noble fill gas (e.g., argon or helium), creating electron-ion pairs. Under an applied electric field, these charges drift toward the electrodes—with electrons collected at the anode—generating an electrical pulse that indirectly registers the neutron. The electrode structure commonly employs two coaxial cylinders or parallel plates. The fill gas must remain stable at high temperatures to prevent decomposition, while the insulators are critical for maintaining electrical isolation between the anode and cathode under high voltage.

As the energy of fission fragments produced by nuclear reactions is much higher than the energy of secondary particles produced, it has a stronger ionization capability, with a larger output current than ionization chambers, and is less affected by γ-rays, making it suitable for high γ-ray environment applications.

To achieve neutron flux measurements spanning several orders of magnitude, high-temperature fission chambers typically operate in three distinct modes: pulse mode, Campbell mode, and current mode.

The pulse mode is suitable for low neutron flux levels. In this regime, the detector treats each fission event as an individual electrical pulse, and the neutron flux is directly determined by counting the number of pulses per unit time. By setting an amplitude discrimination threshold, pulses from low-amplitude gamma-ray backgrounds can be effectively rejected, endowing this mode with excellent signal-to-noise ratio and gamma discrimination capability. As the neutron flux increases, pulse signals begin to overlap, necessitating a transition to the Campbell mode. In this mode, individual pulses are no longer resolved; instead, the fluctuation of the output current is measured. According to the Campbell theorem, the mean square value of this fluctuating signal is proportional to the product of the square of the charge per pulse and the average pulse rate, thereby establishing a proportional relationship with the neutron flux. A notable advantage of this mode is its strong suppression of the gamma background, as the small charge contribution from gamma-induced pulses results in a significantly lower impact on the overall fluctuation compared to fission fragment pulses. Under very high neutron flux conditions, signal fluctuations become averaged out, and the output stabilizes into a steady ionization current, requiring the use of the current mode. This mode directly measures the average ionization current generated collectively by fission fragments and gamma rays. Although it cannot distinguish between neutron and gamma events and requires background compensation, the current mode remains the only effective approach for high-flux measurements due to the absence of pulse pile-up effects.

**Figure 15:**
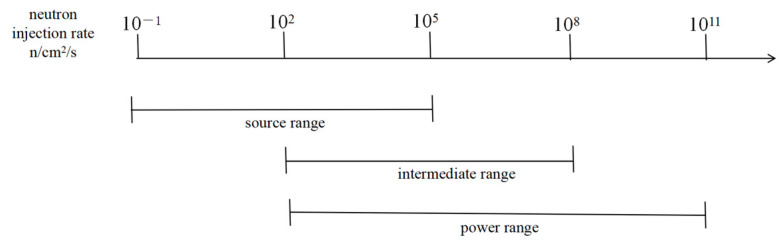
Range of Neutron Injection Rates for Reactors.

High-temperature fission chambers are integral to reactor nuclear measurement systems, which are categorized as either in-core or out-of-core. Out-of-core systems, primarily comprising neutron detectors positioned around the periphery of the reactor pressure vessel, monitor neutron leakage from the core. In-core systems, by contrast, employ detectors installed within the reactor core itself to directly measure the neutron flux. The neutron injection rate in the reactor can generally be divided into three segments according to size as shown in Figure 15: source range, intermediate range and power range. Fission chambers are generally available in the source and intermediate range regions, and some can be designed and optimized to cover higher injection power range regions.

### 4.2. High Temperature Performance Testing and Reactor Monitoring Applications

Facing the demand for neutron flux monitoring in high-temperature extreme environments within the core of fourth-generation nuclear reactors, high-temperature-resistant design of fission chamber detectors has become a key direction for technological breakthroughs. In 2017, a team led by Meng Lingjie at the Chinese Academy of Sciences evaluated a French Photonis CFUE32 fission chamber across a temperature range of 17.1–550 °C [59]. Performance degradation, marked by an anomalous count rate increase and the disappearance of neutron peaks, was observed above 450 °C, revealing the operational temperature limit of conventional designs. In a subsequent advancement, Shunli Qiu and colleagues at the Wuhan Second Ship Design Institute developed a localized fission chamber for ex-core nuclear measurements in 2024 [60]. The device was experimentally validated at two facilities: the horizontal thermal column of the 49-2 swimming pool reactor at the China Institute of Atomic Energy and the reference thermal neutron field at the Chinese Academy of Metrology. Results demonstrated excellent performance, including a high-voltage plateau, high thermal neutron sensitivity, and strong gamma discrimination. Its key metrics meet engineering application requirements, marking a significant step in the localization of this critical technology.

Since the beginning of the 21st century, international research has increasingly focused on enhancing high-temperature adaptability. In 2005, the Japan Atomic Energy Research Institute [61] demonstrated that a miniature fission chamber (Φ 6/12 mm) could monitor neutron fluxes in the range of 1 × 10^16^–10^17^ n/cm^2^/s at 250 °C. The Photonis High-Temperature Fission Chamber (HTFC), developed by Filliatre’s team in France [62], extended the operational temperature range to 400–550 °C (with an outer diameter of 48 mm) and validated its feasibility for deployment above the core in simulations of a Generation IV sodium-cooled reactor. In 2020, Galli et al. [63] innovatively replaced argon with xenium as the filling gas, raising the fission chamber’s temperature tolerance to 400 °C. Catalyzed by the European Union’s Advanced Nuclear Monitoring System program (2013–2020), high-temperature fission chamber technology achieved a breakthrough. Under the leadership of Germany’s Jülich Research Centre, a novel prototype was developed using advanced ceramic composites. This detector is capable of withstanding temperatures up to 700 °C in short-term tests, with a sustained operational limit of 600 °C. The exceptional thermal conductivity and radiation hardness of the composite material effectively mitigate the performance degradation observed in conventional materials under extreme conditions. The team led by Kevin Tsai [64] systematically evaluated the performance of two fission chamber technologies. The Micro-Pocket Fission Detector (MPFD), developed by INL, failed to yield measurable neutron signals at temperatures up to 850 °C, with performance primarily limited by issues in manufacturing hermeticity and electrical noise. In contrast, the miniature fission chambers supplied by the CEA demonstrated superior high-temperature resilience. Specifically, a 3 mm-diameter chamber exhibited an excellent linear response at temperatures up to 700 °C; however, its performance degraded near 750 °C due to a loss of seal integrity. Meanwhile, a 7 mm-diameter high-temperature variant, despite a rated tolerance of 600 °C, could not operate in the desired pulse mode because of excessive cable noise. The study clearly identifies that future designs of high-temperature fission chambers must prioritize robust chamber sealing, employ low-noise cables, and ensure reliable operation in either pulse or Campbell mode to meet the stringent demands of high-temperature and high-radiation environments.

Parallel to research efforts, the commercialization of high-temperature fission chambers has gained significant momentum. In 2022, Kevin Tsai [65] evaluated Photonis CFR43 miniature fission chambers (Φ 3 mm) in a dry tube environment at 350 °C. The ^235^U-based detector demonstrated a calibrated sensitivity of 2354 cps/kW with a temperature drift of only −2.25%. Meanwhile, Reuter-Stokes [66] introduced the RS-C3 series of commercial fission chambers capable of operating at 200–300 °C. Its MIC miniature ionization chamber (Φ5 mm × 50 mm) enables thermal neutron monitoring across 10^11^–10^14^ n/cm^2^/s within an ultra-compact form factor. In the United Kingdom, Ultra Energy [67] achieved a breakthrough by developing a fission chamber rated for 800 °C. In France, EXOSENS [68]—building on the legacy of Photonis and Centronic—is advancing detector operating temperatures beyond 600 °C. Under its small modular reactor development program, the company has made key progress through optimized electrode design, improved metal-ceramic sealing, and novel high-temperature connectors: a modified CFUE model targets 800 °C operation, while the FC152 fission chamber has achieved full functionality at 850 °C in internal tests.

Measures for addressing high-temperature challenges in fission chambers are structured around three key aspects: material selection, structural design, and gas medium composition. Internationally, numerous research groups employ specialized alloys such as Inconel 600 for electrodes and structural components, ensuring the retention of mechanical strength and corrosion resistance under elevated temperatures. High-purity alumina or magnesium oxide ceramics are adopted as insulating materials, providing reliable electrical insulation under high-temperature and intense radiation conditions. Additionally, mineral-insulated metal-sheathed cables are utilized for signal transmission to maintain performance in extreme environments. To mitigate the risk of partial discharge—commonly encountered with conventional argon fill gas at high temperatures—the latest generation of fission chambers has incorporated xenon as the filling medium. Thanks to its superior dielectric strength and higher breakdown voltage, xenon effectively suppresses discharge phenomena, thereby enabling stable detector operation in temperature regimes ranging from 500 °C to 650 °C without compromising neutron detection capability.

These developments have advanced fission chambers beyond laboratory validation, enabling their application in real-time monitoring of high-temperature reactor cores. The operational temperature range of these detectors has been progressively elevated from the conventional limit of 450 °C to 800 °C, facilitating the commercialization of related products. This progress addresses ultra-high temperature application scenarios (>500 °C) that are beyond the current capabilities of diamond and 4H-SiC detectors, as noted earlier, thereby providing critical support for the safety monitoring of advanced nuclear energy systems.

### 4.3. Research on Miniaturized Structure Design and Performance Optimization

**Figure 16:**
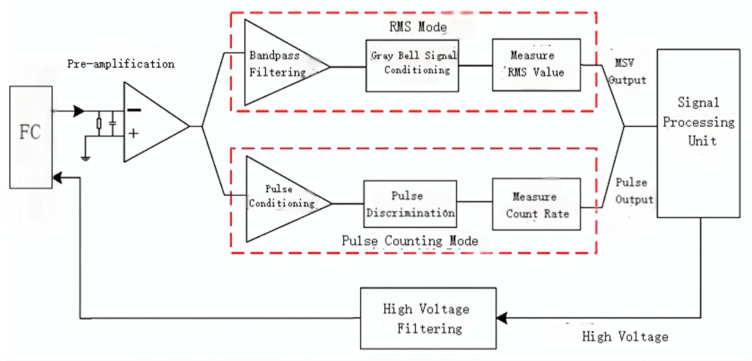
Schematic diagram of detector working mode [71].

In the field of neutron flux monitoring for nuclear reactors, the fission chamber serves as a key neutron detection instrument and represents essential instrumentation for ensuring safe and stable reactor operation. In recent years, research teams worldwide have achieved notable progress in the development of miniaturized fission chambers, wide-range measurement techniques, and performance optimization. In 2022, Zhenyu Yin et al. [69] from the Chinese Academy of Engineering Physics dedicated their efforts to the development of a novel pocket-sized micro fission chamber (MPFD). Building upon the third-generation MPFD originally developed by Kansas State University in the United States, the research team conducted in-depth optimization of the detector structure through detailed electric field simulation studies, achieving a more compact design and enhanced mechanical strength. Furthermore, the selection of 95% alumina ceramic as the insulating material significantly improved the instrument’s high-temperature resistance. This newly developed miniature fission chamber offers the distinctive capability of flexible deployment at any location within the reactor core for real-time neutron flux monitoring, thereby providing an advanced approach for high-precision in-core measurement. Wu et al. [70] at the Northwest Institute of Nuclear Technology used Monte Carlo software to establish an effective method for modeling the neutron sensitivity of miniature fission chambers (MFC). It was found that the MFC neutron sensitivity first increases and then decreases with increasing thickness of the fissile coating, which led to the determination of an optimal solution of 10 μm for the thickness of the fissile layer. Immediately after, in 2024, Chunchi Wang’s team at the State Nuclear Demonstration Power Station LLC [71] carried out a systematic verification of the performance of the developed fission chambers in terms of wide-range neutron injection rate. Its experimental results clearly show that the output characteristics of this fission chamber can effectively meet the linearity indexes required for practical engineering applications in both pulse operation mode and Campbell operation mode, as shown in Figure 16, which proves its reliability in full-range monitoring. In the following year, Jiayu Sun et al. [72] of the China Academy of Atomic Energy Sciences further deepened the study of wide-range measurements in fission chambers. Their innovative fusion of three current signals generated by fission chambers in pulse mode, Campbell mode and current mode. Using advanced digital signal processing algorithms, the seamless connection and smooth transition of the three key measurement intervals, namely, source range, intermediate range and power range, are successfully realized on Matlab and FPGA platforms. These successive research breakthroughs have significantly improved the comprehensive performance of fission chamber detectors and provided stronger technical support for the safe operation and condition monitoring of nuclear reactors. Żerovnik et al. [73] performed high-resolution in-core measurements in a TRIGA reactor using a miniature fission chamber manufactured by CEA. By employing a precision positioning system, they obtained axial profiles of the neutron and gamma fields with a spatial resolution of 1 mm, and systematically characterized the linear dynamic range of the detector in both pulse and current modes. Experimental results demonstrated that the fission rate distribution measured in pulse mode showed excellent agreement with MCNP simulations, providing strong experimental validation for the accuracy of the computational model. The study further confirmed that an array of absolutely calibrated in-core fission chambers could form the basis of an online power monitoring system with superior accuracy compared to conventional ex-core instrumentation, highlighting the significant potential of fission chambers in enhancing reactor monitoring capabilities.

### 4.4. Short

Regarding high-temperature performance, research efforts have been dedicated to extending the operational limits of conventional fission chambers. Through optimization of filling gases (e.g., the adoption of xenon), along with innovations in materials and manufacturing processes, the operating temperature range of the new generation of fission chambers has been substantially expanded—from a previous upper limit of approximately 450 °C to 550 °C or even 800 °C. This enhancement enables their application in high-temperature and extreme environments, such as within the core of fourth-generation sodium-cooled fast reactors, thereby addressing the technological gap in ultra-high-temperature neutron monitoring scenarios.

In the area of miniaturization and performance optimization, technological progress has focused on reducing detector dimensions and achieving wide-range measurement capabilities. Refined electric field simulations and structural designs have led to the development of more compact and mechanically robust miniature fission chambers, which can be flexibly deployed inside reactor cores. Furthermore, by integrating signals from pulse, Campbell, and current operation modes and applying advanced digital signal processing techniques, seamless and full-range neutron flux monitoring—from reactor startup to full power—has been successfully realized. This achievement markedly improves measurement reliability and linearity.

In summary, synergistic innovations in high-temperature design, structural miniaturization, and intelligent measurement modes have led to comprehensive enhancement of fission chamber detector performance. These advances provide critical technical support for the safety monitoring and refined management of advanced nuclear energy systems.

## 5. Self-Powered Neutron Detector

### 5.1. Working Principle

Self-powered neutron detectors (SPNDs) are widely employed for in-core neutron flux measurement in third-generation nuclear power plants, owing to their simple structure, compact size, long service life, and exceptional resistance to high temperatures and irradiation [74]. These intrinsic advantages make them particularly suitable for the harsh in-core environments characterized by intense radiation and elevated temperatures. It serves a vital function in nuclear reactor monitoring, physics experiments, and radiation protection, enabling informed safety management and power regulation of reactors through reliable data acquisition.

**Figure 17:**
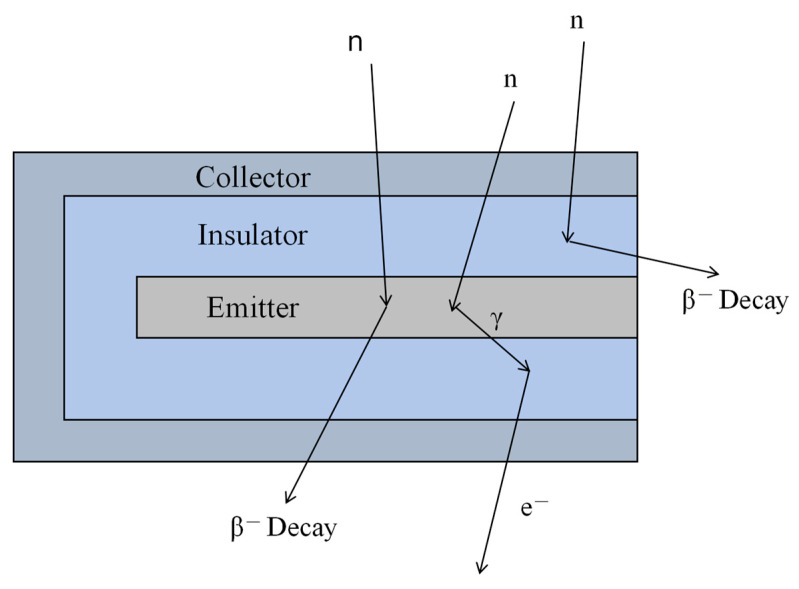
Schematic of the basic structure of a self-powered neutron detector.

A typical SPND consists of the following key components: the emitter, made of materials like rhodium, cobalt, or silver that undergo neutron-induced nuclear reactions; the insulator, typically composed of MgO or Al_2_O_3_; and the collector, which may use materials such as stainless steel [4]. The overall structure is shown in Figure 17. and includes three parts: the emitter, the collector and the insulator. The neutron incidence first enters the emitter, which under the action of neutrons undergoes a nuclear reaction to emit electrons and becomes an anode. The electrons generated in the emitter have a high energy and can escape from the emitter material and enter the insulating layer and subsequently reach the collector pole. The collector pole attracts electrons to become the cathode, creating a potential difference through the insulating layer. An external wire is drawn from the emitter to form a current signal proportional to the incident neutron injection rate [75]. The term “self-powered” refers to the capability of measuring the emitter current directly, without requiring an external bias voltage.

According to their current response characteristics, self-powered neutron detectors can be categorized into two main types: delayed and prompt. In delayed SPNDs, neutron capture by the emitter produces intermediate unstable nuclides alongside gamma-ray emission. These nuclides then undergo beta decay with a relatively long half-life, generating electrons that contribute to a slowly rising, delayed current signal. In contrast, prompt SPNDs also involve neutron capture and gamma-ray emission. However, the gamma rays immediately produce electrons via the photoelectric effect, resulting in an instantaneous current signal. This prompt response occurs on a timescale that is negligible for practical measurement purposes [76].

### 5.2. Detector Test Validation and Engineering Applications

The development of self-powered neutron detectors (SPNDs), which are essential for in-core neutron flux monitoring, is largely driven by the evolving requirements of reactor engineering. The ongoing development of Generation III and IV nuclear energy systems has driven a significant evolution in SPND technology. Its application scope has expanded from traditional in-core detection in thermal reactors to ex-core monitoring, as well as to the challenging environments of fast and fusion reactors. This progression has been accompanied by parallel advances in experimental validation, engineering applicability, and simulation capabilities.

With the maturation of its experimental validation framework, SPND technology has seen a significant expansion in its engineering scope, enabling its full-scale deployment in third-generation pressurized water reactors like the CAP1400 and Hualong One. Li Shucheng’s team at the State Nuclear Power Technology Corporation reported successful in-pile testing of 12 domestic vanadium SPNDs in the CARR reactor, achieving a sensitivity deviation of 1.68%, superior to the ≤2% design specification [77]. In 2020, Huang Youjun et al. from the Nuclear Power Institute of China described a comprehensive optimization of a rhodium SPND for Hualong-1, focusing on its thermal neutron sensitivity, non-neutron noise, and physical dimensions [78]. The detector was validated in the CAER 49-2 reactor, with results confirming that it fully meets the core’s neutron flux measurement requirements and possesses the necessary engineering adaptability for integration with reactor internals and fuel assemblies. SPNDs have demonstrated unique value in the field of severe accident monitoring. Within the DISCOMS project, Barbot et al. [79] developed an innovative SPND system designed to monitor corium relocation in pressurized water reactors. A key aspect of their research involved the use of the MATiSSe simulation toolkit, a multi-step Monte Carlo computational tool developed by CEA, which enables precise prediction of SPND responses in mixed radiation fields. The project innovatively integrated a rhodium-based SPND, characterized by its high thermal neutron sensitivity, with a platinum-based SPND, which responds to both neutrons and gamma rays, within a single instrumented string. Simulation results revealed that during a severe accident, the current from the rhodium SPND drops sharply due to the rapid decrease in neutron flux, while that from the platinum SPND rises significantly under the intense gamma field resulting from corium relocation. These opposing trends in detector signals provide a clear diagnostic criterion for tracking accident progression, offering a robust technical solution for the remote and reliable ex-vessel monitoring of corium movement.

Simultaneously, the application of SPNDs has expanded beyond traditional thermal neutron reactors to encompass more advanced nuclear energy systems. In seminal studies, the research groups led by Vasudha Verma [80] and Loïc Barbot [81] pioneered the use of TRIPOLI-4/DARWIN software (TRIPOLI-4 and DARWIN 2.3) for multiphysics coupling simulations. Their work addressed the optimization of prompt emitter materials to enhance the neutron/gamma signal ratio, providing a crucial assessment of SPND feasibility for fast reactor applications. In 2017, V. Verma et al. [82] from Uppsala University, Sweden, demonstrated that a platinum (Pt) SPND can enable dynamic monitoring of the core power in a sodium-cooled fast reactor (SFR) at full-power operation. However, they also noted that the measurable power range of this configuration is limited and proposed that a comprehensive radial monitoring system, integrating SPNDs with peripheral fission chambers, would be necessary for full coverage.

In a parallel development concerning ex-core detection, the team of Mohd Ali [83] pioneered the application of a vanadium SPND in an ex-core fault detection system. The thermal neutron flux measured experimentally was consistent with simulation results in terms of order of magnitude, thereby verifying the detector’s feasibility for this role. A key challenge identified, however, is the delayed response characteristic of the detector, which requires further improvement to meet the demands of real-time monitoring.

In the field of fusion energy, Prasoon Raj et al. [84] introduced an innovative flat-plate sandwich SPND design to overcome the limited sensitivity of conventional detectors for fast neutron measurements. To address this bottleneck, their design offers a larger effective sensitive area, a compact form factor, and greater flexibility than traditional cylindrical detectors. This design provides a practical solution for online neutron flux monitoring within the confined spaces of the ITER Tritium Breeding Module (ITER-TBM).

### 5.3. Emitter Material Selection and Performance Study

The pursuit of advanced emitter materials and optimized structural designs is critical to overcoming the performance barriers of SPNDs. This development is essential to meet the demanding specifications of fourth-generation reactors and fusion devices for superior radiation tolerance, gamma resistance, and effective fast neutron response. The evolution toward Generation IV reactor systems has shifted the application of SPNDs from thermal reactors, dominated by thermal neutrons, to fast reactors and fusion devices characterized by fast neutron spectra and intense gamma backgrounds. Consequently, the requisite material properties for detector emitters have become increasingly demanding and environment-specific. As early as 2007, the team of M. Alex et al. [85] pioneered the use of Inconel as an emitter material. This low-atomic-number material significantly reduces gamma sensitivity and improves the neutron-to-gamma signal ratio, with experimental confirmation of its long service life and low cost, making it particularly suitable for high-gamma environments. However, the detector exhibits relatively lower neutron sensitivity compared to conventional cobalt and platinum SPNDs.

In 2014, M. Angelone’s team [86] systematically evaluated the limitations of commercial SPNDs for fast neutron monitoring in the ITER-TBM. They identified the inadequacy of traditional emitters due to their low cross-section for fast neutrons and proposed new candidate material systems, including Be, Na, Al, Cr and Ag. This work has outlined a direction for developing SPNDs adapted to fast neutron spectra. In a collaborative study between the Belgian Nuclear Research Centre (SCK·CEN) and CEA, Vermeeren et al. [87] developed and tested multiple SPNDs. For γdetection, a bismuth-emitter tubular configuration (BiTub) demonstrated optimal performance, achieving approximately 50% higher sensitivity than the standard design, along with rapid response and excellent long-term stability. In neutron detection, Co, V and Rh-emitter SPNDs featuring a continuous sheath design were subjected to a three-week irradiation test in the BR2 reactor. Results indicated that all detectors exhibited favorable linear response and low signal-to-noise ratio. Among them, the Rh-based detector showed the highest sensitivity, while the Co-based variant displayed pronounced prompt response characteristics, albeit with greater susceptibility to gamma interference.

In a 2025 study, Sipeng Du et al. [88] from Xi’an Jiaotong University simulated the neutron/gamma field and detector response in an SFR using the Geant4 and SPND Signal simulation toolkits. By systematically comparing the current characteristics, signal composition, and burnup behavior of V and Hf emitters, they demonstrated for the first time the combined advantages of hafnium in long-term operation. The study also revealed a dependence of detector response on its in-core position, underscoring the importance of position-specific correction in SFR neutron monitoring.

The consistency of SPND performance is highly dependent on its manufacturing process. Addressing this, researchers from Lanzhou University [89] employed advanced modeling to quantify the sensitivity contributions of various process parameters in rhodium SPNDs. Their analysis identified the thickness of the insulating layer as the principal determinant of sensitivity, while factors such as rhodium wire eccentricity and variations in trace element content were found to have a negligible impact. This insight provides a direct theoretical basis for optimizing process tolerance control. Collectively, these advancements are pushing SPNDs beyond the performance limits of traditional rhodium and vanadium systems, thereby laying a crucial material and design foundation for neutron monitoring in next-generation ultra-high-temperature fast and fusion reactors.

### 5.4. Optimization of SPND Model Construction

A profound understanding of the response mechanisms and accurate sensitivity calculations form the foundational basis for achieving high-precision, online neutron flux monitoring in nuclear reactors using SPNDs. Recent years have witnessed significant breakthroughs in the construction and correction of SPND theoretical models. In 2021, the team led by Daibo Yang [75] conducted an in-depth analysis of the dynamic response mechanism of rhodium-based SPNDs. Through theoretical investigation, they attributed the signal delay primarily to the element’s radioactive half-life. Furthermore, the team systematically compiled a compendium of technical methods for signal delay compensation, providing a comprehensive comparison of their correction efficacy, advantages, and limitations. This work offers valuable guidance for engineering applications.

Several research teams have proposed innovative solutions to the problem of sensitivity prediction bias due to the simplification of traditional models: Lepore et al. [90] systematically validated the response model of SPNDs across both thermal and fast neutron energy spectra using MCNPX Monte Carlo simulations. The validation results indicate that, although quantitative discrepancies exist between the simulated values and the manufacturer’s thermal neutron data as well as experimental measurements from the TAPIRO fast reactor, the simulations successfully reproduced the current signals within the correct orders of magnitude. This outcome confirms the reliability of the simulation methodology as an effective tool for SPND design. An accurate theoretical model of vanadium SPND signal current and thermal neutron sensitivity was developed by Wu et al. [91] at the University of Science and Technology of China. The computational results demonstrated excellent consistency with experimental data, thereby establishing a solid physical foundation for the quantitative application of vanadium detectors in fast and fusion reactors. The Tsinghua University [92] developed a generalized sensitivity assessment model founded on Monte Carlo simulation. To this end, they successfully reproduced the burnup evolution of the detector throughout a commercial pressurized water reactor (PWR) fuel cycle via a normalized data-processing workflow, thereby validating its reliability for engineering predictions; The team from Lanzhou University [93] developed a more accurate multiphysics coupling model for rhodium SPNDs, which significantly improved the precision of sensitivity simulations, thereby providing a reliable tool for neutron flux monitoring in nuclear fuel irradiation performance assessment.

These studies have achieved mechanism deepening, algorithm innovation and validation closure through delay correction, sensitivity modeling and stack plant data benchmarking. By addressing the persistent challenge of “model simplification versus actual deviation” in SPNDs, this research facilitates a transition from empirical design toward accurate simulation, thereby providing technical support for neutron monitoring and the safe operation of Generation IV reactors.

### 5.5. Short

SPNDs, as key equipment for neutron flux monitoring in reactor cores, have made significant technological advances in recent years in terms of experimental validation, material development, and modeling, which have boosted their application in advanced nuclear energy systems.

In the context of test validation and engineering applications, SPNDs have been extended from conventional thermal reactors to third-generation pressurized water reactors, including designs such as the CAP1400 and Hualong-1. Domestic vanadium and rhodium SPNDs demonstrate superior performance in terms of sensitivity and interface adaptability. Meanwhile, SPND technology is being progressively applied to advanced nuclear systems such as fast reactors and fusion reactors. Through multi-physics coupling simulations, researchers have verified the feasibility of using SPNDs for power monitoring in sodium-cooled fast reactors, and have proposed the implementation of a robust monitoring system by integrating fission chambers. Progress has also been made in beyond-reactor applications. For instance, vanadium SPNDs are employed for fault detection, although their delayed signal response continues to pose challenges for real-time monitoring. In the field of fusion energy, compact sandwich-structured flat-panel SPNDs have been developed to meet the requirements for neutron monitoring in confined spaces, such as those found in the ITER project.

In pursuit of suitable emitter materials for Generation IV and fusion reactors, researchers have been investigating a range of new materials capable of withstanding their intense fast neutron fluxes and strong gamma radiation fields. Initial efforts focused on using Inconel alloys to mitigate gamma sensitivity. Later studies shifted to materials like beryllium and sodium to enhance neutron sensitivity. On the material front, recent modeling work has demonstrated the multifaceted advantages of hafnium in the long-term operation of sodium-cooled fast reactors. In terms of fabrication, studies have confirmed that the sensitivity of rhodium SPNDs is predominantly governed by the thickness of the insulating layer, a finding that establishes a theoretical basis for precision manufacturing.

To address the challenges of SPND response delay and sensitivity prediction deviations, research efforts have focused on several innovative approaches. These include mechanistic analysis of the dynamic response in rhodium detectors, the development of precise theoretical models for vanadium detectors, the creation of a general-purpose sensitivity assessment tool based on Monte Carlo methods, and the construction of multi-physics coupling models. These advancements have collectively enhanced the simulation accuracy and engineering prediction reliability of SPNDs, marking a transition from empirical design to precise simulation.

In summary, driven by expanded testing, material innovations, and refined models, SPND technology is progressively improving its performance in complex in-core monitoring. These developments collectively enhance its neutron measurement accuracy, serving as a critical enabler for the safe and reliable operation of advanced nuclear systems.

## 6. Summary and Outlook

### 6.1. Summaries

This paper presents a systematic review of the research progress of 4H-SiC detectors, diamond detectors, high-temperature fission chambers and self-supplied-energy neutron detectors operating in high-temperature and high-irradiation environments. In addition to a compendium of its working principles, operating characteristics, and general structure, this work summarizes the domestic and international technological development status, application fields, and current frontier research hotspots for four types of detectors.

#### 6.1.1. Material Properties Determine Application Boundaries

Diamond detectors offer stable performance in sub-300 °C environments, capitalizing on an ultra-wide bandgap and exceptional radiation hardness, yet suffer from performance degradation at elevated temperatures due to carrier transport imbalance. In comparison, 4H-SiC detectors have ascended as the mainstream choice for high-temperature neutron detection, with their operational range pushed to 500 °C by structural innovations like super-junction PIN architectures and optimized HDPE conversion layers. Leading the frontier of ultra-high-temperature operation, fission chambers have surpassed the 800 °C limit through innovations in inert gas fillings and miniaturized designs such as MICs. Meanwhile, SPNDs cement their unique position in real-time core monitoring, relying on advanced emitter materials and enhanced delay correction models to ensure indispensable performance.

#### 6.1.2. The Engineering Process Is Clearly Differentiated

Diamond and 4H-SiC detectors remain in the laboratory-to-engineering transition phase, hindered by the high cost of single-crystal preparation and challenges in controlling epitaxial layer defects. Consequently, global development efforts have primarily focused on testing detectors under simulated conditions in laboratory settings. While some teams have successfully deployed these detectors for neutron flux measurements in real reactor environments, large-scale commercialization has not yet been achieved. Furthermore, research on their application for direct neutron monitoring and reactor power indication in operational reactors remains limited.

Fission chambers and self-contained energy neutron detectors are more maturely developed and commercially available as traditional neutron flux monitoring instruments. Fission chambers for in-core neutron flux monitoring at high temperatures have been successfully developed and commercialized by numerous international teams, with ongoing research focused on miniaturization and wide-range monitoring capabilities. Notwithstanding these advancements, SPNDs remain crucial in the neutron monitoring systems of third-generation nuclear reactors due to their simple construction, long lifespan, and self-powered operation. However, their integration into fourth-generation reactor cores still presents formidable challenges, particularly in achieving miniaturized deployment and ensuring long-term stability under intense irradiation.

#### 6.1.3. Technical Bottlenecks Need to Be Broken Through

All four detector types face common challenges, including signal distortion at elevated temperatures, low sensitivity to fast neutrons, and the accumulation of irradiation damage. Specific issues for each type are as follows: In fission chambers, high temperatures can induce a non-linear increase in count rate and the disappearance of neutron peaks. These phenomena are presumed to stem from a sudden change in ionization efficiency due to the thermal decomposition of filler gases; However, a precise quantitative model for this behavior is yet to be established. For both diamond and 4H-SiC semiconductor detectors, incomplete charge collection constitutes a key limiting factor. At elevated temperatures, the increased thermal excitation of charge carriers enhances their trapping at deep-level defects, leading to reduced charge collection efficiency (CCE) and intensified polarization effects. This results in signal “quenching”—where the measured signal amplitude falls below its theoretical value. Even at room temperature, inherent defects and trapping centers within the material can degrade the effective CCE, adversely affecting the detector’s energy linearity and long-term stability [32]. Therefore, enhancing material purity, employing defect engineering, and optimizing electrode contacts are fundamental approaches to improving detector performance under high-temperature and high-radiation conditions. SPNDs suffer from low response cross-sections for fast neutrons above 1 MeV. While new emitter materials are being explored for Generation IV fast reactors, a critical database of fast neutron reaction cross-sections for these materials is missing. Finally, the efficiency of both diamond and 4H-SiC detectors is constrained by nuclear reaction threshold energies, resulting in low detection efficiencies (<5%) for neutrons in the 0.1–5 MeV energy range.

### 6.2. Future Outlook

A primary challenge is the development of innovative material processes. Optimizing the deposition technology of neutron conversion layers has become a key research focus for enhancing diamond detectors. A critical challenge is achieving atomic-level uniform doping of isotopes like ^6^Li and ^10^B directly into the diamond lattice via advanced chemical vapor deposition. Such integration would circumvent the signal attenuation associated with conventional coating methods and significantly improve thermal neutron detection efficiency. Similarly, for 4H-SiC neutron detectors, the epitaxial layer thickness is a primary factor limiting detection efficiency. Consequently, research efforts are directed at developing novel processes to grow thicker epitaxial layers, thereby expanding the sensitive volume. In the case of SPNDs, the high reaction cross-section of conventional emitters is effective for thermal neutrons, yet this advantage diminishes in reactor environments with a significant share of fast neutrons. Therefore, an urgent priority is the exploration and development of new emitter materials possessing superior response cross-sections for fast neutrons.

Another priority is to investigate behavior under extreme environmental conditions. In response to the development of Generation IV reactor systems, researchers worldwide are increasingly focusing on the performance of neutron detectors under high-temperature conditions. Significant progress has been made by employing high-temperature furnaces, thermocouples, and other heating devices to simulate extreme thermal environments, enabling systematic evaluation of detector performance characteristics. Despite these advances, considerable research gaps remain. Although the four types of neutron detectors discussed in this paper are capable of operating at elevated temperatures, most exhibit significant performance degradation as temperature increases. For instance, diamond and 4H-SiC neutron detectors have reported maximum operating temperatures of up to 500 °C, while fission chambers and SPNDs generally demonstrate even higher thermal limits. The high-temperature performance of these detectors is largely influenced by the material properties of key functional components, including the sensitive area, insulation, packaging, and signal transmission cables. Additionally, there is a growing research emphasis on the behavior of associated electronic readout circuits under high-temperature conditions, which represents a critical direction for future investigation.

## Figures and Tables

**Table 1:** Summary of Performance Parameters for Four Neutron Detectors.

Technical Indicators	Lower Limit of Neutron Flux Detection	Neutron Flux Detection Upper Limit	Maximum Reliability Operating Temperature	Dimensions (Diameter)	Technology Maturity
Diamond detector	Excellent (as low as 10^14^ nV)	General (typically ≤10^10^ nv)	Superior (theoretically ≥600 °C)	Medium	Proof-of- concept stage
Silicon carbide semiconductor detector	Excellent (as low as 10^4^ nV)	Poor (typically ≤10^12^ nv)	Excellent (theoretically ≥800 °C)	Medium	Proof-of- concept stage
High-temperature fission chamber	Excellent (as low as 10^2^ nV)	Excellent (up to 10^14^–10^15^ nv)	Outstanding (about 800 °C)	Excellent (≤1.5 mm)	Mature
Self-powered neutron detector	Poor (typically ≥10^9^ nV)	Excellent (up to 10^15^ nv)	Exceptional (≥1000 °C)	Excellent (≤2 mm)	Quite mature

**Table 2:** Performance comparison of two types of diamond detectors.

Performance Parameters	Single-Crystal Diamond Detector	Polycrystalline Diamond Detector
Charge collection efficiency	Excellent (typically >97%, with a maximum of 98.9%)	Moderate to good (typically 60–90%)
Energy resolution	Excellent (up to 2.1–3.7% for alpha particles)	Poor (typically >10%)
Maximum reliable operating temperature	Excellent (experimentally verified typically ≤ 330 °C, theoretically ≥ 600 °C)	Good (experimentally verified typically ≤ 250 °C)
Neutron detection efficiency	Moderate (depends on conversion layer, e.g., ^6^LiF)	Moderate (depending on transition layer)
Radiation resistance	Exceptional	Excellent
n/γ discrimination capability	Excellent	Moderate
Technology maturity	proof-of-concept stage	Laboratory research and development phase

## Data Availability

The data presented in this study are available in this article.
